# Factors influencing failed vaginal trial of labor and subsequent cesarean section in elderly multiparous women, and maternal and neonatal outcomes

**DOI:** 10.3389/fgwh.2026.1729147

**Published:** 2026-03-09

**Authors:** Nan Feng, Hai gang Zhang, Linna He, Yuju Qin

**Affiliations:** Department of Critical Care Medicine, Shenzhen Nanshan District People’s Hospital, Shenzhen, Guangdong, China

**Keywords:** advanced Maternal Age, cesarean Section, multipara, placenta Previa, premature Rupture of Membranes, trial of Vaginal Delivery

## Abstract

**Objective:**

The rate of failed trial of vaginal delivery (TVD) requiring subsequent cesarean section (CS) is relatively high among elderly multiparous women, which seriously impacts maternal and neonatal health. This study aimed to systematically investigate the factors influencing such conversion to CS in this population and evaluate their effects on maternal and neonatal outcomes, thereby providing evidence for clinical interventions.

**Methods:**

A retrospective analysis was conducted on all multiparous women aged over 35 years who had a history of vaginal delivery only, admitted to the First Affiliated Hospital of Jinan University from January 2022 to December 2023. After screening, the eligible subjects were divided into the vaginal delivery group and the converted cesarean section group. Univariate analysis was used to screen for potential influencing factors, followed by multivariate Logistic regression model to identify independent risk factors for conversion to CS. Meanwhile, differences in maternal and neonatal outcomes between the vaginal delivery group and the converted CS group were compared and analyzed.

**Results:**

Among the 510 elderly multiparous women, 422 achieved successful vaginal delivery, while 88 underwent conversion to CS due to failed TVD. Univariate analysis showed that maternal age and gestational weight gain (GWG) in the converted CS group were significantly higher than those in the vaginal delivery group (*P* < 0.05); there were statistically significant differences between the two groups in the incidence of gestational hypertension, hypothyroidism, and placenta previa (*P* < 0.05). Multivariate Logistic regression analysis revealed that age [OR = 1.113,*P* = 0.021,95%confidence interval (CI): 1.016–1.218], GWG above Institute of Medicine (IOM) guideline (OR = 1.977,*P* = 0.044,95%CI 1.019–3.837), gestational hypertension (OR = 6.903,*P* = 0.000,95%CI 3.127–15.239), Premature rupture of membranes (PROM)(OR = 0.263,*P* = 0.003,95%CI 0.108–0.644), hypothyroidism (OR = .044,*P* = 0.004,95%CI 1.434–6.464), and placenta previa (OR = 8.097,*P* = 0.006,95%CI 1.800–36.415) were all independent risk factors for conversion to CS (*P* < 0.05). The area under the receiver operating characteristic (ROC) curve of the binary Logistic regression model was 0.764 (95% CI: 0.709–0.820), indicating good predictive performance. In addition, the incidences of postpartum hemorrhage, neonatal asphyxia, fetal distress, and neonatal admission to the neonatal intensive care unit (NICU) in the converted CS group were significantly higher than those in the vaginal delivery group (*P* < 0.05).

**Conclusion:**

Conversion to CS due to failed TVD in elderly multiparous women is influenced by multiple factors, including maternal age, excessive GWG, and various pregnancy complications. In clinical practice, optimizing intrapartum management strategies, strengthening prenatal weight management, and enhancing fetal weight monitoring are expected to reduce the rate of conversion to CS and improve maternal and neonatal clinical outcomes.

## Strengthsand limitations of this study

- This study focuses on the specific population of “elderly multiparous women”—a group with a relatively high rate of conversion to CS after failed TVD The targeted selection of subjects addresses the clinical need for exploring risk factors in this high-risk population, making the research results highly targeted.
- Beyond analyzing risk factors for CS conversion, the study also compares differences in maternal and neonatal outcomes [e.g., postpartum hemorrhage, neonatal asphyxia, fetal distress, Neonatal Intensive Care Unit (NICU) admission] between the vaginal delivery group and the converted CS group. This comprehensive outcome assessment provides direct and multi-dimensional evidence for clinical intervention strategies.
- The study may have missed some potential confounding factors, such as the subjects' specific fetal positions and precise estimation of fetal weight during pregnancy.
- Due to the limitation of funding and resources, this study will be only conducted in a tertiary hospital.

## 1 Introduction

Globally, the trend of delayed childbearing age has become increasingly prominent, leading to a continuous expansion of the elderly parturient population. For elderly multiparous women (≥35 years old with a history of childbirth), physiological changes associated with aging and multiple pregnancies make their perinatal management a key challenge in the field of obstetrics (1). Vaginal delivery has always been the preferred mode of delivery recommended by obstetricians, owing to its lower surgical trauma, shorter recovery period, and significantly reduced risk of long-term complications (2). However, the complex pathophysiological characteristics faced by elderly multiparous women—such as decreased uterine myometrial contractility, impaired placental function, and increased risk of fetal growth restriction (FGR)—result in a significantly higher probability of failed TVD requiring subsequent CS (3).

In recent years, a large body of evidence-based medicine has shown that the rate of conversion to CS among elderly multiparous women ranges from 22.9% to 33.3% across different regions (4, 5). This urgent shift in delivery mode not only significantly increases the risk of intraoperative and postoperative complications, including severe postpartum hemorrhage, infection, and organ injury, but also may cause long-term psychological trauma, such as post-traumatic stress disorder (PTSD) (6, 7). For neonates, conversion to CS is closely associated with an increased risk of asphyxia, respiratory distress syndrome (RDS), and admission to the NICU (8).

Although existing studies have established various prediction models for conversion to CS based on maternal and fetal clinical parameters, most of these models focus on young multiparous women, and their applicability to the specific population of elderly multiparous women is extremely limited. Given the uniqueness of elderly multiparous women in terms of fertility decision-making and perinatal management, there is an urgent need to establish an accurate risk assessment system for this group. Through a large-sample retrospective cohort analysis, this study used a binary Logistic regression model to systematically explore the independent risk factors for failed TVD and subsequent conversion to CS in elderly multiparous women, as well as their impacts on maternal and neonatal outcomes. The aim is to provide evidence-based support for clinicians in formulating personalized delivery strategies, thereby improving the quality of perinatal management and enhancing maternal and neonatal health.

## 2 Methodology

### 2.1 Study participants

This was a single-center retrospective cohort study. Data of parturients who delivered at the First Affiliated Hospital of Jinan University between January 2022 and December 2023 were systematically screened. In accordance with the definition of elderly parturients by the International Federation of Gynecology and Obstetrics (FIGO), multiparous women aged ≥35 years with a history of childbirth were included.

Inclusion criteria: ① Singleton pregnancy; ② Cephalic presentation; ③ Term pregnancy (gestational age ≥37 weeks and <42 weeks); ④ Low-risk pregnancy (no severe medical/surgical comorbidities or obstetric complications); ⑤ Multiparous women with all previous deliveries via vaginal birth.

Exclusion criteria: ① Obvious cephalopelvic disproportion (assessed based on pelvic measurement and estimated fetal weight); ② Genital tract malformation precluding vaginal delivery; ③ Elective CS due to social factors; ④ Multiple pregnancies, abnormal fetal presentation, or gestational age <37 weeks or ≥42 weeks; ⑤ Patients with missing data.

This study involves human participants and was approved by the Ethics Committee of the First Affiliated Hospital of Jinan University (Ethics Approval No.: KY-2020-094). All participants provided written informed consent prior to their enrollment in the study.

### 2.2 Data collection

A standardized data collection form was used, and data were extracted from the hospital's electronic medical record system by obstetricians and gynecologists who had received unified training. The collected content included the following:

Demographic data: Occupation, educational background, age, pre-pregnancy body mass index (BMI), obstetric and gynecological history, presence of thalassemia, hepatitis B virus (HBV) carrier status, and glucose-6-phosphate dehydrogenase (G6PD) deficiency (favism).

Antenatal data: GWG, gestational hypertension,Gestational diabetes mellitus (GDM), PROM, Intrahepatic Cholestasis of Pregnancy (ICP), placenta previa, hypothyroidism, polyhydramnios, oligohydramnios, and neonatal weight.

Delivery and neonatal outcomes: Fetal distress, neonatal asphyxia, neonatal weight, admission to the NICU, and postpartum hemorrhage (PPH).

To ensure data accuracy, all data were cross-checked by two independent researchers. In case of discrepancies, a third senior obstetrician and gynecologist made the final decision.

### 2.3 Sample size calculation

According to the calculation formula for estimating the population rate through sampling surveys: *n* = Z2*α*/2 P(1 − P)/*δ*2, assuming an error allowance of *δ* = 0.05, and the rate of conversion to CS among elderly multiparous women ranges from 22.9% to 30.0% with an average of 26.5%, *α* = 0.05, Z0.05/2 = 1.96, and *P* = 0.265, the calculated sample size *n* ≈ 300. Considering the exclusion criteria, a dropout rate of 10% was set, and the sample size was determined to be at least 330.

### 2.4 Data analysis

Statistical analysis was performed using SPSS 27.0 software. Measurement data conforming to a normal distribution were expressed as mean ± standard deviation (±s), and comparisons between groups were conducted using the independent samples *t*-test. Measurement data with a non-normal distribution were presented as median (interquartile range) [M (P25–P75)], and the Mann–Whitney U rank-sum test was used for intergroup comparisons. Categorical variables were described as counts and percentages (%), and the chi-square test (*χ*^2^-test) was applied for intergroup comparisons; when the expected frequency was <5, Fisher's exact test was used instead.

A binary Logistic regression model was constructed to identify independent risk factors for converted CS. Variables with *P* < 0.05 in the univariate analysis were included in the multivariate model, and the stepwise backward elimination method was used for variable selection. The predictive performance of the model was evaluated using the ROC curve, and the area under the curve (AUC) and its 95% CI were calculated. All statistical tests were two-tailed, and a *P*-value <0.05 was considered statistically significant.

### 2.5 Relevant definitions and diagnostic criteria

All clinical diagnoses in this study were based on the latest authoritative guidelines and consensus statements, as follows:

Indications for cesarean section: including medical indications such as fetal distress, cephalopelvic disproportion, and labor arrest (9).

Premature rupture of membranes: Rupture of the fetal membranes before the onset of labor; PROM occurring before 37 weeks of gestation is defined as preterm premature rupture of membranes (PPROM) (10).

Pre-pregnancy BMI classification: Followed the BMI standards for Chinese adults: BMI <18.5 kg/m^2^ was classified as underweight, 18.5–23.9 kg/m^2^ as normal weight, 24.0–27.9 kg/m^2^ as overweight, and ≥28 kg/m^2^ as obese (11).

Gestational weight gain: Based on the 2009 guidelines of the Institute of Medicine (IOM, now part of the National Academies of Sciences, Engineering, and Medicine, NASEM), the recommended GWG ranges for pregnant women with different pre-pregnancy BMI categories were: 12.5–18.0 kg for underweight women, 11.5–16.0 kg for women with normal weight, 7.0–11.5 kg for overweight women, and 5.0–9.0 kg for obese women. GWG below the recommended range was defined as insufficient GWG, and GWG above the recommended range as excessive GWG (11).

Hypertensive disorders of pregnancy (HDP): In accordance with the criteria of the International Society for the Study of Hypertension in Pregnancy (ISSHP), it was diagnosed as a systolic blood pressure ≥140 mmHg and/or diastolic blood pressure ≥90 mmHg, with or without abnormal urine protein (12).

Gestational diabetes mellitus: Referenced the 2023 guidelines of the American Diabetes Association (ADA). GDM was diagnosed if any of the following criteria were met in the oral glucose tolerance test (OGTT) performed between 24 and 28 weeks of gestation: fasting plasma glucose (FPG) ≥ 5.1 mmol/L, 1-hour plasma glucose ≥10.0 mmol/L, or 2-hour plasma glucose ≥8.5 mmol/L.

Postpartum hemorrhage: Blood loss ≥1000 mL within 24 h after delivery, or accompanied by signs and symptoms of hypovolemic shock.

Placenta previa: The lower edge of the placenta is adjacent to or covers the internal os of the cervix after 28 weeks of gestation (13).

Intrahepatic cholestasis of pregnancy: It is an obstetric complication that occurs in the second or third trimester of pregnancy, characterized by pruritus and elevated serum total bile acid (TBA). The diagnosis can be confirmed when the fasting TBA is ≥10 μmol/L or the postprandial TBA is ≥19 μmol/L (14).

Hypothyroidism in Pregnancy:Overt Hypothyroidism: Serum TSH level above the upper limit of the gestational age-specific reference range, and a FT4 level below the lower limit of the gestational age-specific reference range. Subclinical Hypothyroidism: Serum TSH level above the upper limit of the gestational age-specific reference range, with a FT4 level within its normal reference range.

Neonatal asphyxia: spontaneous breathing or failure to establish regular respiration within the first minute after birth, characterized primarily by hypoxemia, hypercapnia, and acidosis as the main pathophysiological changes (15).

Fetal distress: a pathological state in which the fetus is at risk *in utero* due to high-risk factors such as acute or chronic hypoxia and acidosis, manifesting as metabolic disturbances and functional compromise.

## 3 Results

### 3.1 Basic characteristics of study participants and univariate analysis

**Figure 1:**
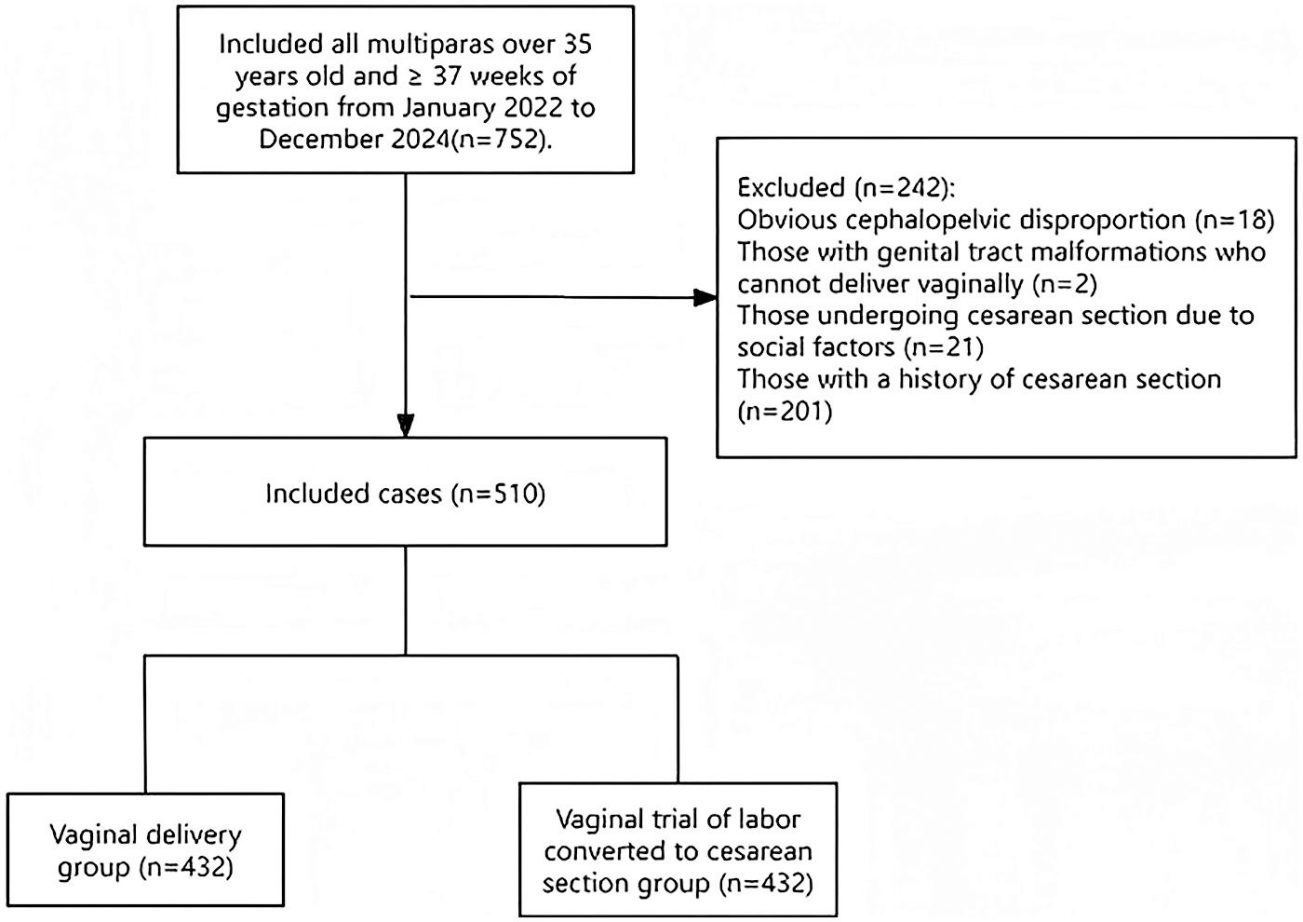
Flowchart of patient enrollment.

After strict screening and exclusion of cases with primary CS, a total of 510 parturients were enrolled in the study cohort (Figure 1). Among them, 422 cases achieved successful vaginal trial of labor (vaginal delivery group) and 88 cases underwent conversion to CS due to failed vaginal trial of labor (converted CS group). The comparison of general data between the two groups showed that there were statistically significant differences in indicators including age, GWG category, hypertensive disorders of pregnancy, PROM, hypothyroidism, and placenta previa (*P* < 0.05). In contrast, no statistically significant differences were observed between the two groups in terms of occupation, educational level, pre-pregnancy BMI category, ICP, history of abortion, GDM, vaginitis, polyhydramnios/oligohydramnios, thalassemia, hepatitis B virus (HBV) carrier status, glucose-6-phosphate dehydrogenase (G6PD) deficiency (favism),and incidence of macrosomia, and low birth weight (LBW) infant (*P* > 0.05). Details are shown in Table 1.

**Table 1:** Comparison of general data between the vaginal delivery group and the converted cesarean section group.

Variable	vaginal delivery group	converted CS group	***t/x^2^***	*P*
	(*n* = 422)	(*n* = 88)		
	*n*/x(%/*μ*)	*n*/x(%/μ)		
Age	37.16 ± 2.34	37.89 ± 2.93	−2.546^a^	0.011
Occupation			0.931^b^	0.334
Employed	336 (79.6%)	66 (75.0%)		
Unemployed	86 (20.4%)	22 (25.0%)		
Education			1.061^b^	0.303
College or below	76 (18.0%)	20 (22.7%)		
University or above	346 (82.%)	68 (77.3%)		
Pre-pregnancy BMI (kg/m^2^)			4.426^b^	0.216
<18.5	42 (10.0%)	6 (6.8%)		
18.5∼23.9	304 (72.0%)	60 (68.2%)		
24.00∼27.9	64 (15.2%)	16 (18.2%)		
≥ 28.0	12 (2.8%)	6 (6.8%)		
GWG			12.364^b^	0.002
Below IOM guidelines	188 (32.7%)	16 (18.2%)		
Within IOM guidelines	158 (37.4%)	30 (34.1%)		
Above IOM guidelines	126 (29.9%)	42 (47.7%)		
ICP			0.064^c^	0.801
No	418 (99.1%)	88 (100.0%)		
Yes	4 (0.9%)	0 (0.0%)		
History of Abortion			2.974^b^	0.085
No	166 (39.3%)	26 (29.5%)		
Yes	256 (60.7%)	62 (70.5%)		
GDM			0.010^b^	0.921
No	290 (68.7%)	60 (68.2%)		
Yes	132 (31.3%)	28 (31.8%)		
Hypertensive Disorders			36.363^b^	<0.001
No	408 (96.7%)	70 (79.5%)		
Yes	14 (3.3%)	18 (20.5%)		
Vaginitis			0.148^b^	0.700
No	406 (96.2%)	86 (97.7%)		
Yes	16 (3.8%)	2 (2.3%)		
PROM			12.071^b^	<0.001
No	324 (76.8%)	82 (93.2%)		
Yes	98 (23.2%)	6 (6.8%)		
Polyhydramnios			0.292^c^	0.589
No	402 (95.3%)	82 (93.2%)		
Yes	20 (4.7%)	6 (6.8%)		
Oligohydramnios			3.391^c^	0.066
No	412 (97.6%)	82 (93.2%)		
Yes	10 (2.4%)	6 (6.8%)		
Thalassemia			0.063^c^	0.802
No	408 (96.7%)	84 (95.5%)		
Yes	14 (3.3%)	4 (4.5%)		
HBV carrier			3.713^b^	0.054
No	388 (91.9%)	86 (97.7%)		
Yes	34 (8.1%)	2 (2.3%)		
Hypothyroidism			9.573^b^	0.002
No	396 (93.8%)	74 (84.1%)		
Yes	26 (6.2%)	14 (15.9%)		
Favism			0.148^c^	0.700
No	406 (96.2%)	86 (97.7%)		
Yes	16 (3.8%)	2 (2.3%)		
Placenta Previa			3.996^c^	0.046
No	418 (99.1%)	84 (95.5%)		
Yes	4 (0.9%)	4 (4.5%)		
LBW infant			0.255^c^	0.614
No	418 (99.1%)	86 (97.7%)		
Yes	4 (0.9%)	2 (2.3%)		
Macrosomia			3.300^b^	0.114
No	384 (93.4%)	86 (97.7%)		
Yes	28 (6.6%)	2(2.3%)		

a For data with a normal distribution, the parametric t-test was employed.

b The chi-square test was used for categorical data analysis.

c When the theoretical frequency in any cell was less than 5, Fisher's exact test was applied instead.

### 3.2 Multivariate logistic regression analysis of converted cesarean section

**Figure 2:**
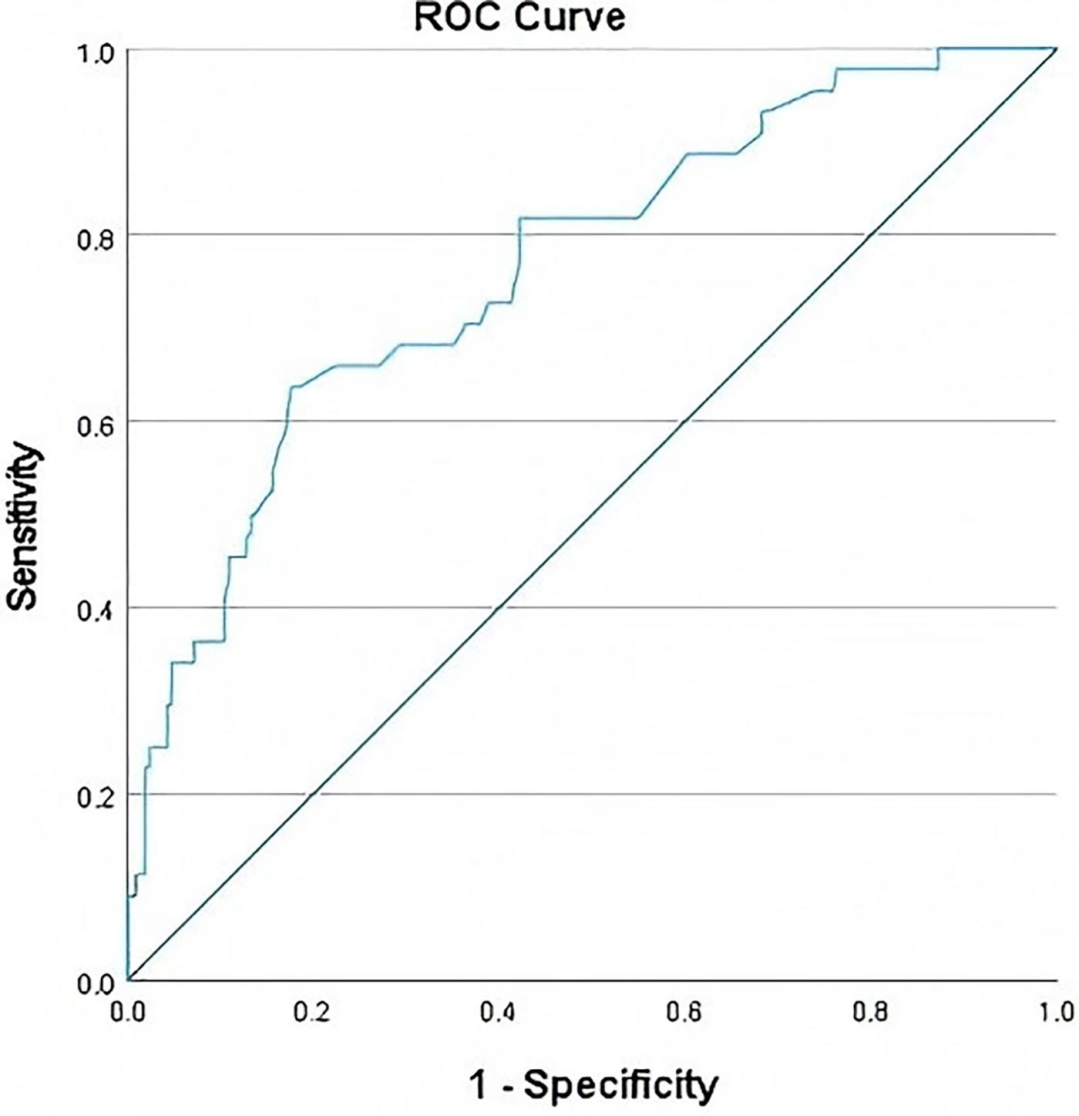
ROC curve.

Variables with statistical significance in the univariate analysis (age, GWG category, gestational hypertension, PROM, hypothyroidism, and placenta previa) were included in the multivariate binary Logistic regression model. Variable assignments are shown in Table 2. The results (Table 3) indicated that for each 1-year increase in age, the risk of converted CS significantly increased OR = 1.113, 95%CI: 1.016–1.218, *P* = 0.021), suggesting that advanced age is a risk factor for converted CS. With normal GWG as the reference, excessive GWG (Category 2) significantly increased the risk of converted CS (OR = 1.977, 95% CI: 1.019–3.837, *P* = 0.044); in contrast, insufficient GWG (Category 1) had no significant effect on the risk of converted CS (OR = 1.285, 95% CI: 0.648–2.551, *P* = 0.473), indicating that excessive GWG is a risk factor for converted CS. Pregnant women with gestational hypertension had a significantly higher risk of converted CS than those without gestational hypertension (OR = 6.903, 95% CI: 3.127–15.239, *P* < 0.001), making it an important risk factor for converted CS. Pregnant women with hypothyroidism also had a significantly increased risk of converted CS (OR = 3.044, 95% CI: 1.434–6.464, *P* = 0.004), suggesting that hypothyroidism elevates the probability of converted CS. Additionally, pregnant women with placenta previa showed a substantially higher risk of converted CS (OR = 8.097, 95% CI: 1.800–36.415, *P* = 0.006), with the most prominent increase in risk among all identified risk factors. Notably, compared with women without PROM, those with PROM had a significantly lower risk of converted CS (OR = 0.263, 95% CI: 0.108–0.644, *P* = 0.003), indicating that PROM is a protective factor against converted CS. To verify the predictive performance of the model, ROC curve analysis was performed on the binary Logistic regression model. The results showed that the area under the ROC curve of the model was 0.764 (95% CI: 0.709–0.820, *P* < 0.001), suggesting that the model has good predictive value for the occurrence of converted CS (see Figure 2).

**Table 2:** Variable assignment table.

Variable names	Assignment methods
converted CS	No = 0; Yes = 1
Hypertensive disorders of pregnancy	No = 0; Yes = 1
PROM	No = 0; Yes = 1
Hypothyroidism	No = 0; Yes = 1
Placenta previa	No = 0; Yes = 1
GWG group	Within IOM guidelines = 0,Below IOM guidelines = 1, Above IOM guidelines = 2

**Table 3:** Results of binary logistic regression analysis for failed vaginal trial of labor and subsequent converted cesarean section in elderly multiparous women.

Variable	B	SE	Wald	*P* value	OR	95%CI
Hypertensive disorders of pregnancy	1.932	0.404	22.867	0.000	6.903	3.127–15.239
PROM	−1.334	0.456	8.566	0.003	0.263	0.108–0.644
Hypothyroidism	1.113	0.384	8.399	0.004	3.044	1.434–6.464
Placenta previa	2.091	0.767	7.433	0.006	8.097	1.800–36.415
GWG group			4.589	0.101		
Below IOM guidelines	0.251	0.350	.515	0.473	1.285	0.648–2.551
Above IOM guidelines	0.682	0.338	4.062	0.044	1.977	1.019–3.837
Age	0.107	0.046	5.337	0.021	1.113	1.016–1.218
Constant	−6.092	1.752	12.091	0.001	.002	

### 3.3 Comparison of maternal and neonatal outcomes

The comparison of maternal and neonatal complications between the two groups showed that there were statistically significant differences in the incidences of postpartum hemorrhage, neonatal asphyxia, and neonatal admission to the NICU (*P* < 0.05); however, no statistically significant difference was observed in the incidence of fetal distress between the two groups (*P* > 0.05). Details are shown in Table 4.

**Table 4:** Comparison of maternal and neonatal complications between the vaginal delivery group and the converted cesarean section group.

Variable	Vaginal Delivery Group	Converted CS Group	*t/x2*	*P*
	(*n* = 422)	(*n* = 88)		
	*n* (%)	*n* (%)		
PPH			11.560^a^	<0.001
No	372 (88.2%)	88 (100.0%)		
Yes	50 (11.8%)	0 (0.0%)		
Neonatal asphyxia			4.690^b^	0.030
No	422 (100.0%)	86 (97.7%)		
Yes (Apgar ≤ 7 score)	0 (0.0%)	2 (2.3%)		
Fetal distress			2.250^b^	0.134
No	416 (98.6%)	84 (95.5%)		
Yes	6 (1.4%)	4 (4.5%)		
Neonatal			10.935^a^	<0.001
Admission to NICU				
No	386 (91.5%)	70 (79.5%)		
Yes	36 (8.5%)	18（20.5%)		

a The chi-square test was used for categorical data analysis.

b When the theoretical frequency in any cell was less than 5, Fisher's exact test was applied instead.

Specifically, the incidence of postpartum hemorrhage in the vaginal delivery group was significantly higher than that in the converted CS group (11.8% vs. 0.0%, *χ*² = 11.560, *P* < 0.001). In contrast, the incidence of neonatal asphyxia in the converted CS group was significantly higher than that in the vaginal delivery group (2.3% vs. 0.0%, *χ*^2^ = 9.629, *P* = 0.002); both cases of asphyxiated neonates in the converted CS group were mild asphyxia (1-minute Apgar score: 4–7 points) and recovered to normal after resuscitation. Additionally, the incidence of neonatal admission to the ICU in the converted CS group was significantly higher than that in the vaginal delivery group (20.5% vs. 8.5%, *χ*^2^ = 10.935, *P* < 0.002).

Regarding fetal distress, the incidence in the converted CS group (4.5%) was higher than that in the vaginal delivery group (1.4%), but the difference did not reach statistical significance (*χ*^2^ = 2.250, *P* = 0.134). Fetal distress in both groups was mainly manifested as abnormal fetal heart rate monitoring, with no cases of severe meconium-stained amniotic fluid. After timely interventions (such as position change, oxygen supplementation, or emergency CS), no severe adverse neonatal outcomes occurred in either group.

## 4 Discussion

The optimal management of TVD in elderly multiparous women, a distinct subgroup in obstetric practice, remains a subject of significant importance in perinatal medicine (16, 17). With the global trend of delayed childbearing and the implementation of multi-child policies, the number of elderly multiparous women is steadily increasing. A failed TVD, necessitating intrapartum cesarean delivery, not only elevates the risk of surgical complications for the mother but may also adversely impact both the short- and long-term health of the neonate, making it a subject of escalating concern (12, 18). Based on clinical data from 510 elderly multiparous women, this study systematically analyzed the independent risk factors for intrapartum cesarean section and the differences in maternal and neonatal outcomes. The following discussion interprets our findings in the context of recent scientific advances, delving into the potential mechanisms, clinical implications, and limitations of our work.

### 4.1 Mechanisms and clinical interpretation of risk factors for intrapartum cesarean delivery

#### 4.1.1 Non-modifiable risk factors: the risk effects of advanced age and placenta previa

This study confirmed that advanced maternal age is an independent risk factor for intrapartum cesarean delivery (OR = 1.113, 95% CI: 1.016–1.218), indicating an 11.3% increase in the risk of failed TVD for each additional year of age. This finding aligns with the several large-scale cohort studies (1, 3). From a physiological perspective, advanced age is associated with decreased elasticity of uterine smooth muscle, a higher incidence of uterine atony, and a greater likelihood of unfavorable cervical ripening. These factors collectively contribute to prolonged or arrested labor, ultimately necessitating cesarean delivery (3). It is noteworthy that the median age in our cohort was 37 years. Unlike previous studies that broadly defined “advanced maternal age” as ≥35 years, our study specifically focused on the 35–40 year age group, where clinical decision-making is often more contentious. This suggests that even mildly advanced maternal age in multiparous women warrants a more meticulous prenatal assessment of uterine functional reserve (e.g., via ultrasound monitoring of myometrial blood flow).

Placenta previa demonstrated the highest risk magnitude among all identified factors (OR = 8.097, 95% CI: 1.800–36.415), which is directly attributable to the mechanical obstruction it causes. Previous research indicates a success rate of vaginal delivery of less than 10% in women with placenta previa, who are also prone to severe intrapartum hemorrhage. Our study further reveals that in elderly multiparous women, placenta previa not only significantly increases the risk of failed TVD but may also elevate the risk of uterine rupture, particularly in cases of anterior placenta combined with a scarred uterus (13). Therefore, for elderly multiparous women, we recommend a precise ultrasound evaluation of placental location at 20–24 weeks of gestation. If placenta previa is diagnosed, a follow-up scan at 32–34 weeks is crucial to rule out placenta accreta spectrum, thereby informing the timing and planning of cesarean delivery.

#### 4.1.2 Modifiable factors: the clinical management implications of GWG, HDP, and hypothyroidism

Excessive GWG (OR = 1.977, 95% CI: 1.019–3.837) emerged as the most clinically actionable risk factor identified in this study. According to IOM guidelines, elderly multiparous women with a normal pre-pregnancy BMI are recommended to limit their GWG to 11.3–15.9 kg. However, only 34.1% to 37.4% of the participants in our study adhered to this range, and the group with excessive GWG had a significantly elevated risk of failed TOL. Mechanistically, excessive GWG may lead to fetal overgrowth (although the incidence of macrosomia showed no significant intergroup difference in our cohort) and increased adiposity in the maternal pelvic soft tissues, thereby raising the risk of cephalopelvic disproportion and obstructed labor (19, 20). Studies have shown that (21) personalized nutritional guidance (e.g., a low-glycemic-index diet combined with moderate exercise) for elderly multiparous women could increase the rate of GWG compliance to over 60% and improve the success rate of TVD by 23%. This finding strongly suggests that implementing a structured weight management plan prenatally, ideally through a multidisciplinary team (MDT) involving obstetricians and nutritionists, could be a pivotal strategy for reducing the rate of intrapartum cesarean delivery (22).

The significant risk effects of hypertensive disorders of pregnancy (HDP) (OR = 6.903, 95% CI: 3.127–15.239) and hypothyroidism (OR = 3.044, 95% CI: 1.434–6.464) underscore the critical importance of the standardized management of medical comorbidities during pregnancy. The primary mechanism linking HDP to failed TVD involves elevated blood pressure causing reduced placental perfusion, predisposing the fetus to intolerance of labor and increasing the mother's susceptibility to preeclampsia, often necessitating urgent delivery. In our study, the rate of intrapartum cesarean delivery was 20.5% among women with HDP, markedly higher than the 3.3% observed in those without HDP, a finding consistent with systematic review evidence (23). For women with hypothyroidism, insufficient thyroid hormone levels can compromise uterine contractility and fetal neurological development (24). Our results, indicating an almost threefold increase in the risk of failed TVD among women with hypothyroidism, highlight the necessity of preconception and antenatal monitoring of thyroid function, with a treatment goal of maintaining TSH levels below 2.5 mIU/L.

#### 4.1.3 Protective factor: the “dual-effect” controversy of prelabor rupture of membranes

This study found that PROM was an independent protective factor against cesarean section conversion (OR = 0.263, 95% CI: 0.108–0.644), which is inconsistent with the conclusions of some studies suggesting that PROM increases the cesarean section rate. An in-depth analysis of the underlying reasons revealed that this discrepancy may be closely associated with the inclusion criteria, clinical management strategies and intervention measures adopted in the present study, as detailed below. First, this study strictly excluded cases with PROM who failed to initiate labor for more than 12 h and all PROM cases complicated with infection, which effectively avoided passive cesarean section induced by elevated infection risk or labor arrest. Second, active clinical interventions were implemented for all included parturients with PROM, including standardized antibiotic prophylaxis (e.g., penicillins) and continuous fetal heart rate monitoring, which significantly reduced the risk of adverse outcomes such as intrauterine infection and fetal distress. Third, PROM itself can promote cervical ripening and shorten the latent phase of labor, thereby improving the success rate of trial of vaginal delivery (25).

Notably, the conclusion of this study that PROM acts as a protective factor against cesarean section conversion carries a potential risk of misinterpretation. As shown in the data of Table 1, the incidence of PROM was higher in the vaginal delivery group, which suggests the possibility of reverse causality or selection bias in the study results. These findings should therefore be interpreted cautiously in combination with clinical practice, and conclusions should not be drawn solely based on statistical results.

In addition, the dual effects of PROM warrant vigilance. For cases with PROM at a gestational age of <34 weeks, although such cases were not included in this study, previous research has demonstrated that parturients with preterm premature rupture of membranes undergoing trial of vaginal delivery have a higher incidence of neonatal respiratory distress syndrome, necessitating a careful weighing of maternal and neonatal benefits and risks (10).

Therefore, for elderly multiparous women complicated with PROM in clinical practice, comprehensive assessment and individualized decision-making should be conducted based on gestational age, fetal heart rate monitoring findings, infection markers (e.g., C-reactive protein, procalcitonin) and the overall condition of the parturient. Clinicians should not only fully consider the positive effects of PROM and the importance of standardized intervention as identified in this study, but also remain alert to the potential risks of bias and adverse outcomes, so as to avoid one-sided interpretation of the study conclusions.

### 4.2 Clinical implications and management optimization based on maternal-neonatal outcome disparities

#### 4.2.1 Maternal outcomes: the “mode-of-delivery paradox” in postpartum hemorrhage

This study revealed a seemingly paradoxical phenomenon: the incidence of postpartum hemorrhage was significantly higher in the vaginal delivery group (11.8%) than in the intrapartum cesarean section group (0.0%). Notably, in the methodology of this study, the quantification of PPH-related bleeding was performed using a quantitative measurement approach rather than visual estimation, which ensures the accuracy and reliability of the bleeding volume data presented herein.

Interpreting this finding requires a nuanced understanding of the physiological characteristics inherent to each delivery mode. The primary causes of PPH following vaginal delivery include uterine atony, lacerations of the soft birth canal, and retained placental tissues. In contrast, during a cesarean section, surgeons can proactively and directly employ interventions such as uterine artery ligation or intrauterine balloon tamponade to achieve rapid hemostasis (4, 10). However, it is crucial to recognize that while the intrapartum cesarean group demonstrated a lower risk of PPH, they face potential risks associated with surgical complications (e.g., surgical site infection, intestinal adhesions), which were not captured in this study due to limited follow-up duration and warrant further investigation.

From a clinical practice standpoint, this finding underscores a critical strategy: For elderly multiparous women with high-risk factors for PPH (such as multiple gestation or a history of prior uterine surgery), even when a trial of labor is pursued, meticulous preparedness is mandatory. This includes having blood transfusion protocols readily available and ensuring access to interventional radiology (e.g., arterial embolization catheters). Furthermore, intensified monitoring of uterine contractions (e.g., via an intrauterine pressure catheter) is recommended to facilitate the early identification and management of uterine atony (26).

#### 4.2.2 Neonatal outcomes: the association of intrapartum stress with asphyxia and NICU admission

The rates of neonatal asphyxia (2.3% vs. 0.0%) and NICU admission (20.5% vs. 8.5%) were significantly higher in the intrapartum cesarean delivery group. The core reason underlying this disparity is the “intrapartum stress associated with a failed trial of labor.” On one hand, prolonged labor can lead to fetal hypoxia, thereby increasing the risk of asphyxia. On the other hand, the shorter preparation time for an emergency cesarean section may result in the neonatal resuscitation team being less than fully prepared, potentially compromising the efficacy of resuscitation. In this study, both cases of neonatal asphyxia in the intrapartum cesarean group were mild and recovered following resuscitation, underscoring the critical importance of timely intervention. Delaying the decision for cesarean delivery risks the progression to severe asphyxia, which could increase the potential for long-term neurological impairment.

To optimize neonatal outcomes, we recommend establishing clear termination thresholds for trial of labor in elderly multiparous women. Cesarean delivery should be promptly initiated under the following conditions: ① Arrest of the active phase (cervical dilation < 0.5 cm/hour for over 4 h); ② Persistent non-reassuring fetal heart rate patterns, such as late decelerations or significant variable decelerations lasting ≥60 s; ③ Maternal development of preeclampsia or symptoms of heart failure (27). Furthermore, a dedicated neonatal resuscitation team should be readily available for all elderly multiparous women undergoing a trial of labor, ensuring that neonates delivered by cesarean section receive immediate and standardized resuscitation care (28).

### 4.3 Study limitations and future directions

#### 4.3.1 Limitations

Despite employing rigorous statistical analyses, including binary logistic regression and the Hosmer-Lemeshow goodness-of-fit test to control for potential confounding factors, this study has several limitations. First, its single-center design may limit the generalizability of the findings. As the study population was primarily drawn from a tertiary hospital, the results may not fully reflect clinical realities in primary or secondary care settings. Second, the study did not incorporate dynamic intrapartum factors, such as contraction frequency and the rate of fetal descent, which have been demonstrated by multiple studies to significantly influence the outcome of a trial of labor. Third, the follow-up period for maternal and neonatal outcomes was relatively short, precluding the assessment of long-term indicators such as childhood neurodevelopment and maternal pelvic floor dysfunction.

#### 4.3.2 Future research directions

Building upon the identified limitations, future research should focus on the following areas. First, conducting multicenter, prospective cohort studies that enroll elderly multiparous women from hospitals of different levels would help expand the sample size and validate the identified risk factors. Second, employing machine learning algorithms (e.g., Random Forest, deep learning) to integrate dynamic intrapartum data could facilitate the development of more accurate predictive models for intrapartum cesarean delivery, thereby enhancing the precision of clinical decision-making. Third, randomized controlled trials are needed to evaluate the effectiveness of interventions such as “personalized weight management programs” and “early cervical ripening interventions” in reducing the rate of intrapartum cesarean delivery. Finally, long-term follow-up studies are essential to analyze the impact of different delivery modes on the lifelong health of both mother and child, thereby providing crucial evidence for the “whole-of-life health” framework.

## 5 Conclusion

Based on clinical data of 510 elderly multiparous women, this study systematically identified the independent risk factors, protective factors, and disparities in maternal and neonatal outcomes associated with conversion to cesarean section following TVD. It provides targeted evidence for optimizing clinical delivery management strategies in this population.

The study confirmed that advanced age, placenta previa, excessive GWG, hypertensive disorders of pregnancy, and hypothyroidism are core risk factors for intrapartum cesarean section conversion. In contrast, PROM acts as an independent protective factor under standardized clinical management. Among these, excessive GWG stands out as a modifiable key factor. This finding suggests that personalized weight management implemented by a multidisciplinary team consisting of obstetricians and dietitians—such as low-glycemic index (low-GI) diet combined with moderate physical activity—can effectively improve the success rate of TVD. Additionally, standardized control of gestational comorbidities [e.g., blood pressure management for HDP, maintenance of thyroid-stimulating hormone (TSH) levels below 2.5 mIU/L in patients with hypothyroidism] serves as a critical safeguard for reducing the risk of TVD failure. Analysis of maternal and neonatal outcomes revealed that although the vaginal delivery group had a higher incidence of postpartum hemorrhage, the converted cesarean section group exhibited significantly increased risks of neonatal asphyxia and admission to the ICU. This mode of delivery paradox highlights the necessity for clinicians to dynamically balance maternal and neonatal safety during labor and establish a scientific threshold for terminating trial of vaginal delivery.

A comparison between the findings of this study and previous literature indicates that the risk effects of advanced age, placenta previa, and HDP on cesarean section conversion are consistent with conclusions from multiple large-sample cohort studies and systematic reviews, which verifies the reliability of our results. However, the protective role of PROM differs from the findings of some studies. This discrepancy may stem from variations in inclusion criteria and clinical management strategies, suggesting that clinical decision-making should be tailored to specific diagnostic and treatment scenarios rather than relying solely on the conclusions of individual studies.

The practical implications of this study are reflected in three key aspects. First, it provides a quantitative basis for prenatal risk stratification in elderly multiparous women. By integrating ultrasound findings (placental location, uterine muscle blood flow), laboratory indicators (TSH levels, blood pressure), and medical history data, high-risk individuals can be accurately identified. Second, it clarifies the management pathways for modifiable factors.

Measures such as personalized nutritional guidance and standardized treatment of gestational comorbidities are highly clinically feasible, offering practical solutions for reducing the rate of cesarean section conversion. Third, it proposes a clinical reference standard for the threshold for terminating trial of vaginal delivery, providing practical guidance for timely decision-making during labor and minimizing adverse maternal and neonatal outcomes.

## Data Availability

The datasets presented in this study can be found in online repositories. The names of the repository/repositories and accession number(s) can be found in the article/Supplementary Material.
